# Refractory Gilles de la Tourette Syndrome—Many Pieces That Define the Puzzle

**DOI:** 10.3389/fneur.2020.589511

**Published:** 2020-12-18

**Authors:** Natalia Szejko, Adam Lombroso, Michael H. Bloch, Angeli Landeros-Weisenberger, James F. Leckman

**Affiliations:** ^1^Division of Neurocritical Care & Emergency Neurology, Department of Neurology, Yale School of Medicine, New Haven, CT, United States; ^2^Department of Neurology, Medical University of Warsaw, Warsaw, Poland; ^3^Department of Bioethics, Medical University of Warsaw, Warsaw, Poland; ^4^Child Study Center, Departments of Psychiatry, Pediatrics and Psychology, Yale University, New Haven, CT, United States

**Keywords:** Gilles de la Tourette syndrome, treatment-refractoriness, severe tics, psychiatric comorbidities, tics

## Abstract

Gilles de la Tourette syndrome (GTS) is a childhood onset neuropsychiatric disorder characterized by the presence of motor and vocal tics. The clinical spectrum of GTS is heterogeneous and varies from mild cases that do not require any medical attention to cases that are refractory to standard treatments. One of the unresolved issues is the definition of what constitutes treatment-refractory GTS. While for some other neuropsychiatric disorders, such as obsessive–compulsive disorder (OCD), a clear definition has been established, there is still no consensus with regard to GTS. One important issue is that many individuals with GTS also meet criteria for one or more other neurodevelopmental and neuropsychiatric disorders. In many individuals, the severity of these comorbid conditions contributes to the degree to which GTS is treatment refractory. The scope of this paper is to present the current state-of-the-art regarding refractory GTS and indicate possible approaches to define it. In closing, we discuss promising approaches to the treatment of individuals with refractory GTS.

## Introduction

Gilles de la Tourette syndrome (GTS) is a neuropsychiatric disorder in which both motor and vocal tics coexist. According to the Diagnostic and Statistical Manual of Mental Disorders (DSM-5) (1), to diagnose GTS the following criteria should be fulfilled: the presence of two or more motor tics and at least one vocal tic, although they might not occur at the same time; tics should be present for at least a year and must begin before the age of 18; finally, the symptoms cannot be attributed to administration of drugs or another medical condition, such as encephalitis, Huntington disease, or seizures. In the majority of cases, psychiatric comorbidities are also present, the most frequent being obsessive–compulsive disorder (OCD), attention-deficit hyperactivity disorder (ADHD), depression, and self-injurious behaviors (SIB) (2). Although many patients experience only mild or moderate symptoms, some cases are severe and treatment refractory. One indicator of severe GTS is the level of tic severity measured with the Yale Global Tic Severity Scale (YGTSS) and other related scales (3, 4). However, many patients with severe tics do not demonstrate high impairment in quality of life; on the other hand, some individuals with mild tics describe their tics as severely impairing. This discrepancy is due to the fact that often one tic can be more impairing than another. For example, isolated coprolalia or self-injurious tic could provoke much more impairment than several tics rated as more severe in the YGTSS. That said, the YGTSS does include an overall impairment scale which directly addresses this concern. However, in many patients, psychiatric comorbidities contribute to the final global impairment. Typical first-line treatment for GTS includes well-established behavioral interventions and pharmacotherapy (5). In those cases where these standard treatments are unsuccessful, a number of experimental approaches such as transcranial magnetic stimulation (TMS), deep brain stimulation (DBS), or cannabis-based medicine (CBM) could be implemented (5–8). Further details of the treatment approach in those cases are discussed in the following sections of the manuscript. Therefore, it remains a matter of debate which criteria are required to diagnose GTS patients as refractory. While a consensus about what constitutes treatment refractoriness for other neuropsychiatric disorders has been established, such as OCD, this is not the case for GTS. The purpose of this paper is to summarize the current evidence regarding what constitutes treatment refractory GTS.

## Toward the Definition of Treatment Refractoriness in GTS

### Lessons From the Field of Pathophysiology

A number of molecular mechanisms have been shown to be involved in the occurrence of GTS. These include dopaminergic neurotransmission (9, 10) as well as glutaminergic (11, 12), serotoninergic (13), GABA-ergic (14–16), or endocannabinoid (17, 18) neurotransmission. In addition, a significant number of genetic variants have also been identified [*SLITRK1* (19–21), *DRD2* (22), *DRD3* (23), *AADAC* (24), *ADORA* (25), *BTBD9* (26), *CNR1* (18), *GDNF* (27), *KCNJ5* (28), *IL1RN* (29), *PNKD* (30), and *HDC* (31, 32), to give only some examples]. The first genome-wide association study (GWAS) (33) in GTS demonstrated a combined genetic background for GTS and OCD. The authors conducted a GWAS in 2723 cases (1310 with OCD, 834 with GTS, 579 with OCD plus GTS/chronic tics) and 5667 controls. Surprisingly, no individual single-nucleotide polymorphisms (SNPs) reached genome-wide significance. On the other hand, GWAS signals were enriched for SNPs associated with variants in brain gene expression. However, polygenic risk score (PRS) analysis predicted only 0.6% of the phenotypic variance. A recently published genome wide association study (GWAS) (34) conducted in 4,819 GTS case subjects and 9,488 controls demonstrated one genome-wide significant locus within *FLT3* on chromosome 13 (rs2504235), although this association was not replicated in the population-based sample. GTS-associated genes were expressed in the dorsolateral prefrontal cortex. Additionally, GTS PRS predicted GTS and tic spectrum disorders. Furthermore, GTS PRS was correlated with worst-ever tic severity and a positive family history of tics. Finally, a number of other factors have also been determined to be involved in GTS pathogenesis, particularly, the presence of autoimmune disorders (35–37) as well perinatal factors (38, 39). It can therefore be concluded that GTS pathophysiology is multifactorial which adds to disease complexity and, in some cases, refractoriness.

### Lessons From Other Disorders

As treatment refractoriness in GTS has not been clearly defined yet, one possible approach to determine methodological framework for the criteria could be to adapt the instruments used in similar disorders, especially from the field of psychiatry.

#### Treatment-Refractory Depression

As mentioned by McIntyre et al. (40), there are no clear criteria for treatment-resistant depression (TRD). Nevertheless, it has been suggested that failure to get remission with two or more adequate antidepressant trials defines TRD (41). A recently published systematic review identified five staging models for TRD (42). Each of the staging models defines minimum dosing and duration of therapy, increasing complexity of treatment modality as a function of treatment resistance, and inefficacy with electroconvulsive therapy (ECT) as the most resistant subgroup in major depressive disorder (MDD).

#### Treatment-Refractory Anxiety

Interestingly, the approach used for TRD follows similar logic to what is used for elaboration of criteria for treatment refractory anxiety. As reviewed by Roy-Berne (43), there are two known causes of treatment resistance: “pseudo-resistance” and true treatment resistance. As the cause of “pseudo-resistance,” the authors indicate lack of sufficient treatment resulting from the selection of the wrong compound, the wrong dosage/treatment regime (44), or lack of compliance. Once pseudo-resistance is excluded, one should then consider the diagnosis of treatment-resistant anxiety. Nevertheless, in a recently published review and meta-analysis by Bokma et al. (44), the authors conclude that there is a lack of consensus on the criteria of treatment-refractory anxiety. In the majority of studies (62.9%), treatment resistance was determined after the first treatment failure. Importantly, in the majority of studies the criterium was failure of pharmacotherapy, while only 29.0% also considered failure of psychotherapy. On the other hand, the exact definition of treatment failure was not provided in the vast majority of the studies. Another issue that should be taken into consideration is the treatment duration, ranging between 4 weeks and 6 months. Upon analysis of the available data, the authors came to the following definition of treatment resistant anxiety: the lack of efficacy of at least one first-line pharmacological treatment and also psychological interventions. What is more, this treatment should be used for an adequate duration of at least 8 weeks.

#### Treatment-Refractory OCD

Despite the adequate treatment with established therapies such as selective serotonin reuptake inhibitors (SSRIs) and psychotherapy, almost 30% of OCD patients suffer from treatment refractoriness (45). As summarized by Bloch and Storch (46), critically important issues include making an accurate diagnosis; optimal delivery of first-line treatments for OCD; and the presence of moderating factors that have the potential to influence treatment delivery and efficacy. To provide a more detailed evaluation of those criteria, we will analyze each of them separately. Primarily, symptoms of OCD should be differentiated from the following symptoms and diseases: complex tics, stereotypies, some elements of autistic spectrum disorder, and repetitive behaviors being part of normal development or ruminations present in depression and psychosis. The second part of the assessment of OCD refractoriness is the evaluation of response to first-line treatments. Patients should have received two SSRIs at an adequate dose for 12 weeks total and at least 8 weeks at the maximum dose. An important issue is that in some cases, lack of tolerance makes it impossible to implement appropriate treatment; however, these patients should be treated as treatment-intolerant and not treatment-refractory. Another important component of OCD treatment is cognitive–behavioral therapy (CBT), which should be used for an adequate period of time with an adequate amount of sessions. Moreover, when possible, compliance to treatment should also be assessed. Importantly, coexisting conditions that often occur together with OCD, such as tics or hoarding, may influence treatment resistance. Finally, sometimes a watchful, waiting approach could be beneficial. The course of OCD is heterogeneous with two major phenotypes emerging chronic and episodic (47). The chronic course indicates symptom persistence which can be waxing and waning nature, but without complete relief from symptoms. In contrast, the main feature of the episodic course is the presence of periods with complete remission and subsequent relapses. In case of refractory OCD, it is therefore highly recommended to actively inquire about disease history and adapt the intervention to the subtype of OCD.

A summary of all the criteria of treatment resistance anxiety, OCD, and depression is shown in Table 1.

**Table 1:** Criteria of treatment refractoriness in OCD, anxiety, and depression.

**Disorder**	**Definition of pharmacological RF**	**Definition of RF to psychotherapy**	**Period of treatment required to diagnose RF**
OCD	Two SSRIs at an adequate dose, the maximum tolerated dose, for an adequate duration of treatment	12–24 weekly sessions of CBT (48)	12 weeks total and at least 8 weeks at the maximal dose (48)
Anxiety	Failure to achieve remission/response after treatment with SSRI or SNRI	5 and 20 weekly or fortnightly sessions, with each session lasting 30 to 60 min (49)	Between 4 weeks and 6 months (43)
Depression	Failure to get remission with two or more adequate antidepressant trials (40, 50)	Not included in the criteria	From 6 weeks to 12 months depending on the stage of RF (40)

*OCD, obsessive–compulsive disorder; RF, refractoriness; SSRI, selective serotonin reuptake inhibitor; SNRI, serotonin and norepinephrine reuptake inhibitors; CBT, cognitive behavioral therapy*.

### Subtypes of GTS as Avenue to Define Treatment Refractoriness

#### Pure GTS and GTS Plus

As shown in aforementioned examples of disorders that often coexist and were demonstrated to share common pathophysiology with GTS (51–53), to determine treatment refractoriness it is vital to divide patients into clinical subgroups based on symptom severity. At present, no clear consensus regarding clinical subgroups in GTS has been established. Below, we distinguish between “pure GTS” and GTS plus (54–57). By definition, pure GTS refers to individuals who have motor and phonic tics in the absence of any comorbid conditions. In contrast, GTS plus refers to individuals who have tics coexisting with at least one other neuropsychiatric condition. Below, we present a review of the articles addressing the topic of clinical variability in GTS, particularly, the division in subgroups.

Kano et al. (58) examined 64 Japanese GTS patients and sought to determine the clinical characteristics of individuals with and without so-called generalized tics, which involved the whole body and/or coprolalia. Not surprisingly, patients with “generalized tics” more frequently demonstrated multiple complex vocal tics than those without “generalized tics.” In line with many other studies, individuals with coprolalia demonstrated a more severe and complex clinical phenotype with higher rates of copropraxia, echolalia, and compulsions. As expected, this group was also more socially impaired. On the contrary, those who did not have coprolalia or “generalized tics” were categorized as having a mild phenotype. The authors concluded that coprolalia and “generalized tics” seem to be additional indicators of clinical severity.

Another factor analysis was done by Alsobrook and Pauls (59). They included 85 GTS probands and conducted agglomerative hierarchical symptom clustering. As a result, four clusters were identified: predominantly aggressive symptoms, pure motor and/or phonic tics, compulsive phenomena, and tapping, and the last group characterized by the absence of grunting.

Robertson and Cavanna (56) conducted a principal component factor analysis for members of a large GTS pedigree who had symptoms of the GTS spectrum. They carried out cluster analysis on the pedigree members which consisted of 85 family members, 69 of whom demonstrated the following symptoms: tics, obsessive–compulsive symptoms (OCS) and ADHD. As such, they included a relatively heterogeneous sample, which may have influenced the results. The authors identified three clinical subgroups: the first group, which contained “pure GTS” cases; the second group, comprised of patients chiefly who suffered from ADHD and aggressive behaviors; and the third group, which consisted of patients with “depression–anxiety–obsessional symptoms and self-injurious behaviors.”

In consideration of the previously mentioned findings, it has been also suggested that there is a clinical continuum between pure GTS and GTS plus phenotypes. As mentioned by Grados and Mathews in their review (60), this continuum could be further expanded: GTS with comorbidities would be the most severe and rare form, while transient tics and chronic tic disorders would be the mildest forms. The authors highlight the following phenotypes in the GTS spectrum: pure GTS, GTS+OCD, and GTS+OCD+ADHD.

In 2015, de Haan et al. (61) also sought to establish clinical subgroups within GTS. To carry out this analysis, they analyzed data from 494 patients with GTS from the USA and the Netherlands as well as 351 Dutch family members. As a result of their analysis, they identified three clinical groups: the first group, in which patients predominantly had complex vocal tics and obscene behavior; the second group, in which patients mainly had body tics; and the third group, in which patients mainly had head and neck tics.

Subsequently, Eapen and Robertson (55) compared samples of pure GTS and GTS plus. The authors included in their analysis the following clinical characteristics: disease severity, accompanying clinical features, and family history. To determine symptom severity, they used the following clinical measurements: The National Hospital Interview Schedule (NHIS) for GTS and related behaviors, the Diagnostic Confidence Index (DCI), and the Yale Global Tic Severity Scale (YGTSS). They included 400 patients altogether, but information on psychiatric comorbidities was only available in 222 cases, 13.5% of which had pure GTS. In the GTS plus group, 39% suffered from coprolalia, yet this symptom was not reported by any of the pure GTS patients and this difference was statistically significant. Similarly, the presence of copropraxia was higher (15.4%) in the GTS plus group as compared to the pure GTS (6.3%) group. Interestingly, the only other significant difference between the two groups was that pure GTS was not associated with any family history of OCD, confirming that both groups, pure GTS and GTS plus, may differ specifically when it comes to underlying pathophysiology. Surprisingly, no differences were detected regarding disease severity as measured by the YGTSS and the DCI total score, although results from the DCI approached statistical significance.

In 2016, Sambrani et al. (62) analyzed the clinical characteristics of a large sample (*n* = 1,032) of German patients with tic disorders. The authors found that GTS+OCD was a more severe form of GTS and that comorbid OCD/OCS, depression, and anxiety were part of the GTS spectrum, while ADHD should be treated as a separate problem. They also compared groups of patients with and without specific complex tics (coprophenomena, echophenomena, and paliphenomena), which was distinguished as “full blown GTS.” Particularly, they identified a significant association between coprophenomena, echophenomena, paliphenomena, and comorbidity score. Furthermore, copro-, pali-, and echophenomena were associated with age. Finally, coprophenomena were also correlated with a wide repertoire of psychiatric comorbidities, such as SIB, depression, or rage attacks. All in all, these findings underline that the presence of copro-, pali-, or echophenomena is an indicator of a more severe and complex GTS plus phenotype. Importantly, the same group repeated the previously mentioned efforts by Robertson and Cavanna (56) and attempted to establish subclassification of GTS. In order to accomplish this, they divided the participants into three clusters, as previously mentioned, but were able to classify only one third of them. The vast majority of participants were included in the third category (GTS and comorbid OCD, OCS, anxiety, depression, and SIB). The authors found significant differences between the groups with respect to copropraxia, being significantly more frequent in cluster 2 than in cluster 3. Moreover, tic suppression was more common in the pure GTS group than in cluster 2 (91.7 vs. 78.2%). Finally, the percentage of patients with premonitory urges was significantly higher in cluster 3 than in cluster 1.

In 2017, Pringsheim evaluated the data of 114 Canadian children with GTS (63). Her work focused on examining whether the presence of comorbid ADHD or OCD has an influence on tic severity and treatment. Children with OCD demonstrated significantly higher tic severity. Although children with ADHD were more likely to be treated for their tics within the first 2 years of diagnosis, they did not present with worse tic severity. As such, the author concluded that the association may be related to greater overall psychosocial impairment in children with this comorbidity. Once again, these findings speak to the idea that the presence of a comorbid psychiatric disorder is a clear distinguishing feature of more severe clinical phenotype in GTS.

In a recent paper by Müller-Vahl et al. (64), the authors point out that although chronic tic disorders and GTS are described separately in classifications, there is no clear genetic and phenotypic background for such distinction. In order to tackle this clinical conundrum, the authors analyzed and compared the clinical data of 1,018 patients with GTS and chronic tic disorder. Patients differed in tic severity, with chronic motor tic disorder (CMTD) patients having lower mean tic severity; prevalence of complex motor tics, copropraxia, and echopraxia; and a markedly lower comorbidity score as compared to GTS patients. These findings suggested that both disorders exist along a symptom severity continuum of which GTS constitutes a more severe and CMTD a less severe form. It was therefore suggested to use the term “tic spectrum disorders,” instead of using distinctive diagnostic categories.

#### Subtypes of GTS Based on Longitudinal Studies

In this context, it is worth mentioning a number of studies which examine the course of GTS in the long-term follow-up. One important longitudinal analysis was published by Groth et al. (65), in which they investigated the clinical course of tics and comorbidities trying to establish trajectories of possible clinical phenotypes and their predictors. The authors examined a large clinical cohort recruited at the Danish National Tourette Clinic during the periods 2005–2007 and 2011–2013. They included 314 participants aged 5–19 years at baseline and, 6 years later, 227 participants who took part in the subsequent follow-up visit. At each time point, exhaustive clinical evaluation was conducted. As expected, tic symptoms as well as comorbid OCD and ADHD symptoms declined over time. With time, the clinical phenotype generally evaluated toward pure GTS. They also identified several predictors for the clinical course of GTS in adolescence and early adulthood. Particularly, childhood tics, OCD severity, and ADHD severity predicted the persistence of respective diagnoses in the future. Furthermore, psychiatric comorbidities were found in only 63% of cases at follow-up, while almost 37% of patients had pure GTS.

An important contribution evaluating the course of tics and comorbidities in the perspective of clinical variability was published by Bloch et al. (66). The authors examined the adulthood outcome of tics and OCD symptoms in children with GTS. They included forty-six children with GTS, who had received a structured clinical evaluation prior to age 14, and conducted an expert rating of tics and OCD symptoms at baseline and at a follow-up interview after 7.6 years (in average). The majority of cases (85%) reported a reduction in tic symptoms in the follow-up evaluation, although higher levels of tic severity in childhood were associated with higher levels of tic severity at follow-up.

#### Between Clinics and Genetics—Approach to Refractoriness Based on the Genetic Variability

Taken as background findings from the area of genetics and genomics, it has been established that tics, OCD, and GTS share a common genetic architecture (33, 34, 67). At the same time, the merge between genetic and clinical characteristics may enable the identification of possible subgroups in GTS. Hirschritt et al. (68) showed that social disinhibition is a heritable subphenotype in GTS. The study included 3,494 individuals (1,191 GTS patients and 203 of their first-degree relatives). Heritabilities of the subtypes were estimated and associated with clinical characteristics. The authors conducted exploratory factor analysis as well as latent class model analysis and grouped participants in the following categories: unaffected, simple tics, intermediate tics without social disinhibition, intermediate with social disinhibition, and high rates of all tic types. Across models, a phenotype characterized by high rates of social disinhibition emerged. The presence of social disinhibition was associated with increased odds of comorbid psychiatric disorders, earlier age at GTS onset, and increased tic severity. Darrow et al. (69) identified two heritable endophenotypes for GTS. The authors included 3,494 individuals with GTS, OCD, and ADHD. They carried out symptom-level factor and latent class analyses in GTS families as well as in 882 healthy individuals. The authors were able to identify two cross-disorder phenotypes: symmetry (symmetry, evening up, checking obsessions; ordering, arranging, counting, writing–rewriting compulsions, repetitive writing tics) and disinhibition (uttering syllables/words, echolalia/palilalia, coprolalia/copropraxia, and obsessive urges to offend/mutilate/be destructive). Moreover, both phenotypes were highly heritable. Also, PRS related to GTS were associated with symmetry, while OCD PRS were associated with the symptoms of disinhibition.

A summary of reports investigating different GTS subgroups and longitudinal studies is shown in Table 2.

**Table 2:** Summary of studies investigating subcategories of GTS.

**Study**	**Methodology/aims**	**Population included**	**Main findings**
**SUBGROUP/CLUSTER ANALYSIS**			
Kano et al. (58)	Comparison of patients with and without “generalized tics” and coprolalia	64 GTS patients	• Patients with “generalized tics” demonstrated more frequently multiple complex vocal tics • Individuals with coprolalia demonstrated more severe and complex clinical phenotype with higher rates of copropraxia, echolalia and compulsions • Those who did not have coprolalia or “generalized tics” were categorized as mild phenotype • Coprolalia and “generalized tics” are indicators of clinical severity
Alsobrook and Pauls (59)	Factor analysis, agglomerative hierarchical symptom clustering	85 GTS patients	They identified four clinical groups: • With predominantly aggressive symptoms • With pure motor and/or phonic tics • With compulsive phenomena and tapping • Absence of grunting
Robertson and Cavanna (56)	Principal component factor analysis, cluster analysis	85 family members, 69 demonstrated tics, OCS and ADHD	The following groups were identified: • Pure GTS • Group with patients predominantly suffering from ADHD and aggressive behaviors • Depression–anxiety–obsessional symptoms and SIB
Eapen and Robertson (55)	Comparison of pure GTS and GTS plus	400 patients with tics	• 13.5% of patients had pure GTS • In the GTS plus group, 39% suffered from coprolalia, while no pure GTS patients had coprolalia • The presence of copropraxia was higher (15.4%) in the GTS plus group as compared to the pure GTS (6.3%) group • No differences were detected between pure GTS and GTS plus regarding disease severity
De Haan et al. (61)	Establish clinical subgroups within the GTS phenotype	494 patients with GTS, 315 family members	They identified three clinical groups: • With predominantly complex vocal tics and obscene behavior • With mainly body tics • With mainly head and neck tics
Hirschritt et al. (68)	1) Evaluation of heritable subphenotypes in GTS 2) Heritabilities of the subtypes were estimated and associated with clinical characteristics	1,191 GTS patients and 2003 of their first-degree relatives	• The following groups were identified: unaffected, simple tics, intermediate tics without social disinhibition, intermediate with social disinhibition, and high rates of all tics types • Across models, a phenotype characterized by high rates of social disinhibition emerged. The presence of social disinhibition was associated with increased odds of comorbid psychiatric disorders, earlier age at GTS onset, and increased tic severity • Social disinhibition is a heritable subphenotype in GTS
Sambrani et al. (62)	Comparison of pure GTS and GTS plus	1,032 of patients with tics	• GTS+OCD is more severe form of GTS • Comorbid OCD/OCS, depression, and anxiety were part of the GTS spectrum, while ADHD should be treated as a separate problem • There was a significant association between coprophenomena, echophenomena, paliphenomena, and comorbidity score • Copro-, pali-, and echophenomena were associated with age • Coprophenomena were also correlated with a wide repertoire of psychiatric comorbidities
Darrow et al. (69)	Identify heritable endophenotypes for GTS	3,494 individuals with GTS, OCD, and ADHD	• Two cross-disorder phenotypes emerged: symmetry, disinhibition • Both phenotypes were highly heritable • PRS related to GTS were associated with symmetry, while OCD PRS were associated with the symptoms of disinhibition
Pringsheim (63)	Examine whether the presence of comorbid ADHD and OCD have influence on tic severity and treatment	114 children with GTS	• Children with tics and OCD demonstrated significantly higher tic severity • Children with ADHD were more likely to be treated for their tics within the first 2 years of diagnosis, but did not present more severe tic severity • Presence of comorbidity is a clear distinguishing feature of more severe clinical phenotype
Müller-Vahl et al. (64)	Comparison of chronic tic disorder and GTS	1,018 patients with GTS and chronic tic disorder	• Patients differed in tic severity, with CMTD patients having lower mean tic severity; prevalence of complex motor tics, copropraxia, and echopraxia; and a markedly lower comorbidity score than GTS
			• These findings suggested that both disorders exist along a symptom severity continuum of which GTS constitutes a more severe and CMTD a less severe form. • It was therefore suggested to use the term “tic spectrum disorders”, instead of using different diagnostic categories
**LONGITUDINAL STUDIES**			
Bloch et al. (66)	Evaluate the course of tics and comorbidities	46 children with GTS	• The majority of patients (85%) reported a reduction in tic symptoms in the follow-up evaluation • Only increased tic severity in childhood was associated with increased tic severity at follow-up
Groth et al. (65)	1) Investigate clinical course of tics and comorbidities 2) Establish trajectories of possible clinical phenotypes and their predictors	• 314 participants aged 5–19 years at baseline • 227 participants, who took part in subsequent follow-up visit	• Both symptoms of tics as well as comorbid OCD and ADHD declined over time • With time, clinical phenotype generally evaluated toward pure GTS • Childhood tics, OCD, and ADHD severity predicted the persistence of respective diagnosis in the future • Psychiatric comorbidities were found in only 63% of cases at follow-up, while almost 37% of patients had pure GTS

*Studies are listed in chronological order*.

*GTS, Gilles de la Tourette syndrome; OCS, obsessive–compulsive symptoms; ADHD, attention deficit hyperactivity disorder; SIB, self-injurious behaviors; OCD, obsessive-compulsive disorder; PRS, polygenic risk score; CMTD, chronic motor tic disorder*.

## Definitions of Refractory GTS

### The Concept of Pseudo-Refractoriness

Similar to anxiety or OCD, pseudo-refractoriness should also be considered in GTS (70). Kious et al. also describe this term as “apparent refractoriness” (70). The main reasons for this condition are nonadherence, misdiagnosis, preponderance of psychiatric comorbidities, inadequate treatment (low doses or wrong selection of agent by the clinician), incorrect diagnosis, lack of tolerance, or lack of access to all available therapies. Nonadherence in GTS can affect a significant number of patients. Importantly, compliance is highly dependent on the type of medication administered and its adverse events. For typical antipsychotics (71), this rate can be as high as 78%. It is therefore crucial to rigorously assess the accuracy of the diagnosis. In case of treatment refractoriness, misdiagnosis should also be taken into consideration. Tics could be mistaken with functional movement disorder, myoclonus, chorea, paroxysmal movements, epilepsy, stereotypies, or psychiatric symptoms, for example, compulsions or, rarely, hyperactivity in ADHD. Another factor to consider when facing treatment resistant cases is that uncontrolled, coexisting, neuropsychiatric conditions can also exacerbate tics. Consequently, reevaluation of possible comorbid conditions should be considered. Moreover, the majority of medications used for treatment of tics are associated with significant risk of adverse events and, therefore, are not well-tolerated. In the recently published meta-analysis by Yang et al. (72), the authors established that even 95% of patients treated with neuroleptics experience adverse events and between 30 and 80% discontinue treatment due to lack of tolerance.

### Refractoriness to Pharmacological Treatment in GTS

Taking into consideration phenotypic and genetic variability of GTS, the most severe clinical phenotype also could be interpreted as being refractory. Nevertheless, as mentioned previously, there are no widely established criteria for refractory GTS. Discussion about criteria of refractoriness was raised by the European Society for the Study of Tourette Syndrome (ESSTS) (73). In the ESSTS Guidelines (73), it was suggested that the following clinical criteria indicate refractoriness and, as a consequence, could be qualified for experimental treatment with deep brain stimulation (DBS): (a) tics should last at minimum 5 years, (b) tics should be severe for at least 1 year, and (c) tic severity should be rated ≥35 according to YGTSS. Treatment refractoriness could be established after unsuccessful or not well-tolerated therapy with three different drugs including both typical and atypical neuroleptics in adequate dosages over an adequate period of time. If possible, behavioral therapy for at least 12 sessions should also have been implemented. Moreover, the authors indicate that haloperidol, being the only drug licensed for treatment of GTS in the majority of European countries, suggests that failure of this drug already implicates treatment resistance. The International Deep Brain Stimulation Database and Registry Study Group (74) suggested to identify candidates for DBS in GTS based on the following criteria: lack of response to trials of medications from three different classes, particularly, alpha-adrenergic agonists, dopamine antagonists, and benzodiazepines.

The subgroup of refractory GTS was investigated in only few studies. Colquhoun et al. (75) analyzed records of 329 GTS patients and identified refractory cases using the previously mentioned ESSTS criteria. Only 14 individuals (4.3%) were identified as belonging to the refractory group. Porta et al. (76) systematically reviewed the literature with the aim to revise the criteria of treatment refractoriness in GTS and concluded that this term is poorly defined in the scientific literature. As a matter of fact, the only publications regarding this matter were related to efficacy and tolerability of DBS. They also proposed their own definition of treatment refractoriness, based on their clinical experience. Specifically, they suggested that the following factors should be taken into consideration: no significant improvement in health-related quality of life in response to trials of conventional (typical and atypical neuroleptics) and other pharmacological agents (botulinum toxin or tetrabenazine) as well as resistance to selective serotonin-reuptake inhibitors (SSRI) or tricyclics for comorbid OCD.

Another study by Macerollo et al. (77) reported the findings of the European audit survey. The main goal was to establish tentative criteria for treatment refractoriness in GTS as well as evaluate how frequently these criteria are taken into consideration in routine clinical practice. The methodological approach used in this case included expert-based interviews conducted by seven clinicians with extensive experience in GTS. Different criteria were proposed and rated according to whether the domain was considered as necessary for the definition of refractory GTS. Those criteria that were rated as necessary were also included in subsequent analyses. As a result, the following final criteria were proposed: adequate treatment duration (for at least 3 months); subjective clinical judgment of tic severity as improved or not; change in the YGTSS indicating worsening of tics; reason for the use of maximal dose; and number of single doses missed on average over a 10-day period. Subsequently, seven other experts were asked to comment on the previously selected criteria. This questionnaire was implemented for patients with an unsuccessful trial of at least one anti-tic medication. Two additional criteria were treatment used at the highest dose possible and lack of tolerability. Their final study was conducted on a group of 68 patients rated as treatment refractory; 45 of them were rated as refractory due to lack of efficacy in spite of treatment at maximal dose, while another 23 were included in the refractory group because of intolerability due to side effects. The median time of treatment was of 39 weeks. The final results of this study demonstrated that even among the selected group of GTS experts there was no consensus regarding the treatment refractoriness. While subjectively clinicians did not see improvement under particular treatment, in 39 out of 68 patients, there was a significant tic reduction according to YGTSS total tic score. As also indicated in this paper, not all experts used the YGTSS to confirm refractoriness. It could therefore be concluded that tic severity is not an adequate, unique measurement of treatment refractoriness and should be complemented by the quality of life evaluation. In summary, the authors concluded that they must include lack of tolerability as well as treatment adherence, defined as less than one dose missed on average over a 10-day period, in the refractoriness criteria. A summary of the criteria proposed in this study is found in Table 3, while inclusion criteria for DBS in GTS are demonstrated in Table 4. Importantly, similar criteria were indicated in ESSTS Guidelines (73).

**Table 3:** Criteria of treatment refractoriness for GTS proposed so far.

**Proposed criterium**	**Suggested^**a**^**	**Confirmed after evaluation on patients^**b**^**
Treatment duration (weeks)	Yes	No
Subjective clinical judgment of tic severity as not improved	Yes	No
Change of the YGTSS indicating worsening of tics	Yes	No
Number of single doses missed on average over a 10-day period	Yes	Yes
Unsuccessful trial of at least one anti-tic medication	Yes	No
Treatment used at the highest dose possible	Yes	No
Lack of tolerability	Yes	Yes

GTS, Gilles de la Tourette syndrome; YGTSS, the Yale Global Tic Severity Scale;

a criterium suggested initially by experts;

b *criterium confirmed after evaluation on sample of patients*.

**Table 4:** Criteria for use of DBS in GTS.

**Authors of the criteria**	**Age**	**Minimal tic duration**	**Duration of severe tics**	**Definition of tic severity**	**Definition of PR**	**Definition of refractoriness to BT**
ESSTS (73)	≥18	5 years	1 year	35	1. No effect or lack of tolerance to haloperidol 2. Unsuccessful or not well-tolerated therapy with three different drugs including both typical and atypical neuroleptics in adequate dosages over an adequate period of time	At least 12 sessions
The International Deep Brain Stimulation Database and Registry Study Group (74)	-	-	-	-	No response to trials of medications from three different classes, particularly, alpha-adrenergic agonists, dopamine antagonists, and benzodiazepines	No response to BT
AAN (7)	-			Severe, disabling tics, for example, self-injurious tics	Multiple classes of medication (antipsychotics, dopamine depleters, and α2 agonists)	No response to BT
Porta et al. (76)	-	-	-	-	No response to trials of conventional (typical and atypical neuroleptics) and other pharmacological agents (botulinum toxin or tetrabenazine) as well as resistance to SSRI or tricyclics for comorbid OCD	-

ESSTS, the European Society for the Study of Tourette Syndrome; BT, behavioral therapy; AAN, American Academy of Neurology; SSRI, selective serotonin-reuptake inhibitors; OCD, obsessive–compulsive disorder.

### Treatment of Refractory GTS

As stated above, refractoriness in GTS is highly determined by the response to the evidence-based treatments. In the recently published, evidence-based recommendations by the American Academy of Neurology (AAN) (5, 7), the authors revised the available treatments for GTS. A multidisciplinary panel of experts composed of physicians, psychologists, and patient representatives developed recommendations supported by the systematic review. As a result, they formulated 46 recommendations regarding the assessment and management of tics. They underlined that the treatment should be individualized, consulted on by the patient, family, and the provider. Also, the presence of comorbid conditions should be taken into consideration. Therefore, when deciding on treatment of refractory TS, the first decision should be directed toward identification of the main complaint: tics or comorbidities. The authors of the AAN Guidelines comment on currently available treatments for GTS and mention the level of recommendation, also for the refractory cases. They indicate that where the legislation allows, physicians may consider treatment with cannabis-based medication, a treatment option confirmed to be effective at least for some patients (78–82). In cases of refractoriness, a combination of psychotherapy and pharmacotherapy as well as a combination of different compounds with different mechanism of action could be implemented. In some GTS patients, injecting botulinum toxin locally may alleviate the symptoms of motor or/and phonic tics (83). Additionally, the authors discuss the rationale for the use of the DBS in refractory GTS. They indicate that patients with severe GTS, resistant to medical and behavioral therapy, may benefit from the application of DBS. However, this method should be used only in specialized centers with experience in GTS. Moreover, the patients should undergo a multidisciplinary evaluation by a neurologist or psychiatrist, a neurosurgeon, and a psychologist. Additionally, the benefits must outweigh the risks of the procedure. Clinicians should confirm the diagnosis according to the current DSM-5 criteria and exclude secondary tics. Prior to DBS, multiple classes of medication (antipsychotics, dopamine depleters, and α2 agonists) and behavioral therapy must be administered. Finally, DBS should be used only for severe, disabling tics, for example, self-injurious tics. These recommendations are in line with the European clinical guidelines for Tourette syndrome and other tic disorders. Part IV: deep brain stimulation (73) and the recommendations of the Tourette Syndrome Association International Deep Brain Stimulation (DBS) Database and Registry Study Group (74).

## Conclusions

As demonstrated by the cacophony of findings presented in this review, the definition of treatment refractory GTS still needs to be definitively established. The primary reasons for the lack of an established definition is the robust clinical variability of GTS as well as the fact that it often co-occurs with a number of other disorders that can contribute to the deterioration in quality of life. Future efforts should focus on the collection of large, international clinical data that could enable analysis with machine learning approaches as this could elucidate the presence of objective clinical clusters (84).

## Author Contributions

NS conceived and designed the study, acquired data, and wrote the original draft of the manuscript. NS, AL, JL, MB, and AL-W analyzed and interpreted the data, performed visualization, and reviewed and edited the manuscript. All authors contributed to the article and approved the submitted version.

## Conflict of Interest

JL has received grant and research support from National Institutes of Health. He also serves on the scientific advisory boards of the Brain and Behavior Research Foundation, the European Multicentre Tics in Children Studies, the National Organization for Rare Diseases, Fondazione Child, Empathy for Peace, and the Alliance for Decision Education. He has also received royalties from John Wiley and Sons, McGraw-Hill, and Oxford University Press. MB receives additional contracted research support from Biohaven Pharmaceuticals, Janssen Pharmaceuticals, Emalex Biosciences, and Neurocrine Biosciences. He also receives research funding from the NIH. The remaining authors declare that the research was conducted in the absence of any commercial or financial relationships that could be construed as a potential conflict of interest.
